# Mendelian Randomization Analyses Show an Ambiguous Association between Clonal Hematopoiesis of Indeterminate Potential and Alzheimer's Disease Risk

**DOI:** 10.1002/alz70855_106555

**Published:** 2025-12-24

**Authors:** Matthew H Liu, William S Bush

**Affiliations:** ^1^ Case Western Reserve University, Cleveland, OH, USA; ^2^ Cleveland Institute for Computational Biology, Department of Population and Quantitative Health Sciences, Case Western Reserve University, Cleveland, OH, USA

## Abstract

**Background:**

Clonal Hematopoiesis of Indeterminate Potential (CHIP) is a phenomenon of elevated somatic carcinogenic mutations (measured by variant allele fraction (VAF)) with a paradoxical absence of cancerous phenotypes. CHIP risk increases with age, and is a significant risk factor for future blood cancers and cardiovascular disease, even when controlling for age. Recent analyses show a protective association between CHIP and Alzheimer's disease (AD) using longitudinal and Mendelian Randomization (MR) analyses, which is an unusual finding given age's strong impact on both CHIP and AD.

**Method:**

Using 47 previously identified germline variants that influence predisposition to CHIP, and 21 variants associated with specific CHIP traits, we performed MR with the Kunkle et al. dataset containing 21,982 clinically diagnosed late‐Onset AD non‐Hispanic White (NHW) individuals, and the Bellenguez et al. dataset of 111,326 ‘proxy AD’ NHW individuals. Additionally, using the 1000 Genomes Project Dataset, we calculated polygenic risk scores for CHIP based on APOE genotype to identify relationships between AD predisposition and CHIP risk.

**Result:**

We found no correlation between CHIP and AD risk among the 47 CHIP‐associated variants using either set of AD summary statistics. We also examined the correlation between 21 variants that associate to various CHIP isoforms, which revealed a strong positive association between CHIP and AD risk in both AD datasets. Polygenic risk score data shows an elevated risk for CHIP in those with the APOE3 genotype compared to either the APOE2 or APOE4 genotype, with the risk being more pronounced in those who are homozygous for APOE3 than those who are heterozygous.

**Conclusion:**

Our MR analyses using multiple CHIP categorizations and multiple AD datasets dispute the hypothesis that CHIP is protective against AD, with some findings indicating some CHIP isoforms instead may be an AD risk factor. Our polygenic risk score data further indicates a possible confounding effect of CHIP by APOE genotype which in combination with varying age distributions in many AD datasets, may impact associations reported from previous longitudinal studies. While some CHIP isoforms may indeed be protective for AD, more work is needed to dissect the heterogeneity of CHIP and its relationship to AD risk.

